# Elucidation of ultraviolet radiation-induced cell responses and intracellular biomolecular dynamics in mammalian cells using surface-enhanced Raman spectroscopy†
†Electronic supplementary information (ESI) available: TEM images and UV-vis extinction spectra of AuNCs, time dependent SERS spectra and DF images collected from HSC-3 cells in absence of UV light, additional SERS spectra collected from different cells in their G_1_ phase, DF images collected while UV-C irradiation, Ellman's assay, table showing Raman spectral assignments, video files showing cellular responses under UV-C and UV-A. See DOI: 10.1039/c5sc03817k


**DOI:** 10.1039/c5sc03817k

**Published:** 2015-11-05

**Authors:** Sajanlal R. Panikkanvalappil, Steven M. Hira, Mostafa A. El-Sayed

**Affiliations:** a Laser Dynamics Laboratory , School of Chemistry and Biochemistry , Georgia Institute of Technology , Atlanta , Georgia 30332-0400 , USA . Email: melsayed@gatech.edu; b King Abdulaziz University , Department of Chemistry , Jeddah 22254 , Saudi Arabia

## Abstract

Fingerprinting biochemical changes associated with cellular responses to external stimuli can provide vital information on the dynamics of biological processes and their defense mechanisms. In this study, surface-enhanced Raman spectroscopy (SERS) has been used to elucidate biomolecular dynamics on the response of healthy and cancerous cells towards ultraviolet (UV) light irradiation at the cellular level in real-time. We have identified a number of physiochemical damages to proteins, especially to the chemical structure of the sulfur and aromatic amino acid containing moieties, as well as changes in secondary structure. Furthermore, we found that continuous exposure of short wave UV-C light (254 nm) to living cells can photolytically damage intracellular proteins and can completely arrest nanoparticle transport and trigger apoptosis. However, under similar conditions, this was not observed when the cells were exposed to long wave UV-A light (365 nm). These biomolecular events were probed in real-time using SERS and dark-field (DF) imaging. Specifically, this technique has been utilized for the real-time evaluation of a unique cellular defense mechanism in cancer cells towards UV exposure. Our technique provides a powerful approach to understand the mechanisms of UV light-triggered cell death, protein dynamics, and enhanced cell repair and defense machinery within cancer cells through actively monitoring molecular vibrations.

## Abstract 2

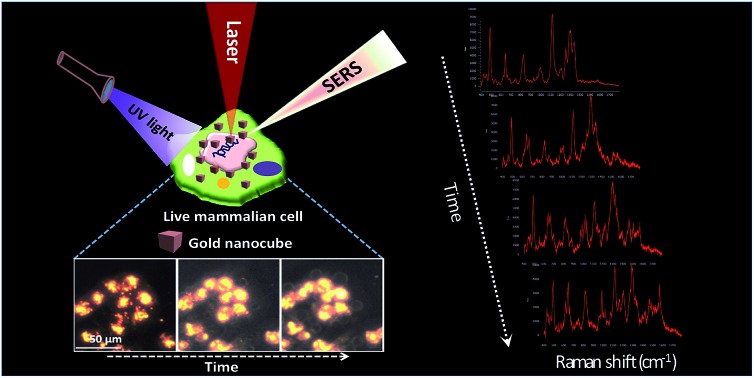
Surface-enhanced Raman spectroscopy has been used to elucidate biomolecular dynamics on the response of mammalian cells towards UV light irradiation.

## Introduction

The real-time visualization of intracellular biomolecular events at the molecular level is of prime importance in biomedical research as it can provide vital information on the mechanism of cellular responses towards various stimuli, which is essential in the development of effective therapeutic strategies.^1^,^2^ Surface-enhanced Raman spectroscopy (SERS) is a very useful experimental approach to this end because it enables noninvasive and continuous monitoring of biochemical processes in real-time.^3^–^10^ Here, we exploit SERS together with plasmonically-enhanced dark-field (DF) imaging to fingerprint the physiochemical modifications of biomolecules inside cancerous and non-cancerous cells in real-time when exposed to ultraviolet-A (UV-A) and ultraviolet-C (UV-C) irradiations.

High-energy UV light is fatal to an array of organisms by damaging their DNA and proteins.^11^–^13^ Previous studies have shown that UV-C (200–280 nm) can potentially damage DNA by inducing strand breaks, bipyrimidine photoproducts, and oxidatively damage bases.^14^–^16^ Though several researchers have proposed various hypotheses on the impact of UV light on protein damage, such as the involvement in the generation of free radicals or reactive oxygen species (ROS) in the modification of protein structures, the details of the mechanisms remain largely unknown. UV photons can induce cellular damage through various mechanisms, such as excitation of cellular chromophores *via* UV light absorption and subsequent chemical reactions, inducing photolytic damage to the biomolecules directly, promote the formation of ROS, *etc.*^17^–^19^ These highly reactive species can interact with cellular macromolecules such as DNA, proteins, fatty acids and saccharides causing oxidative damage to the biomolecules.^19^–^21^ The objective of this study is to establish a molecular fingerprint of the mechanistic pathway in real-time during cell exposure to UV light using plasmonically-enhanced spectroscopic and microscopic techniques. In order to achieve this, we have monitored the cell death mechanisms in cancerous and non-cancerous cell lines while being exposed to UV light for short and long time intervals. The cellular responses to UV irradiation, both intracellular nanoparticle motion and activation of a cellular defense mechanism, were monitored in real-time by using targeted gold nanocube reporters, SERS, and DF microscopy.

## Results and discussion

In order to evaluate the dynamics and physiochemical biomolecular modifications associated with the intracellular biomolecules exposed to UV-C light, we conducted real-time analysis of cellular response from a molecular perspective using SERS. For this study, gold nanocubes (AuNCs) of average diameter 37 nm, conjugated with PEG, RGD and NLS, were used as plasmonic reporters. These nanoparticles were chosen for their strong plasmonic fields and ease of functionalization and were targeted to the nuclear region of HSC-3 cells as reported previously.^22^ The presence of the cell penetrating peptide sequence, RGD, on the AuNC surface facilitates the internalization of the nanoparticles into the cancer cell (HSC-3) *via* receptor-mediated endocytosis.^4^ While the PEG moiety reduces the cytotoxicity of the AuNCs, the nuclear localization peptide sequence, NLS, present on the AuNC surface allows the localization of nanoparticles around the nuclear vicinity.^4^ The TEM image and UV-vis absorption spectrum of the AuNCs are given in Fig. S1.†

**Fig. 1:**
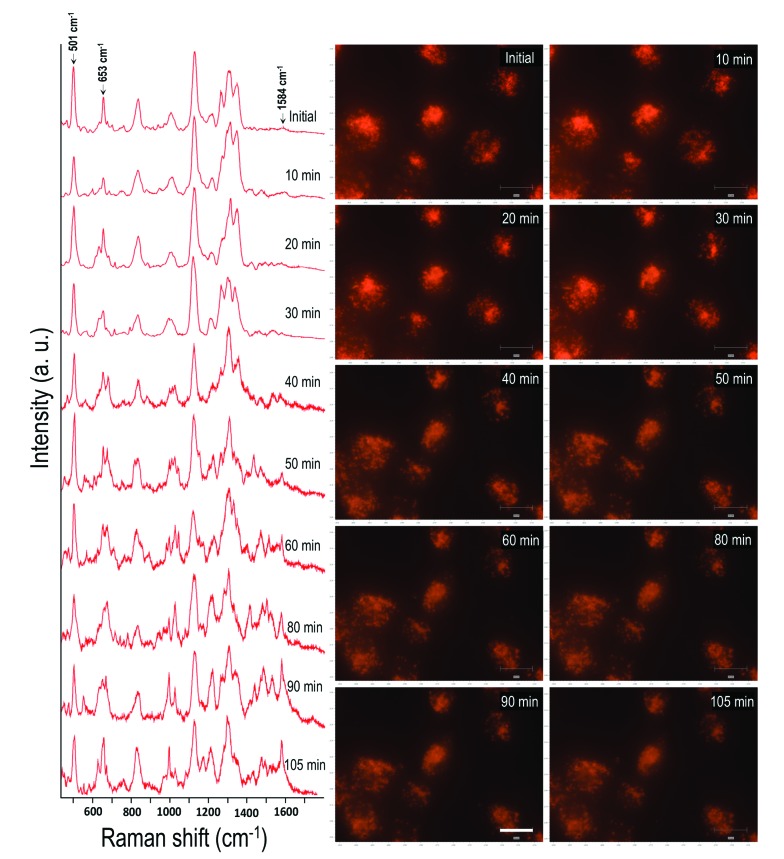
Time dependent SERS spectra collected from a HSC-3 cell while being exposed to UV-C light (254 nm). Corresponding DF microscopy images collected at respective time intervals are also given to observe the AuNCs inside cells. Scale bar is equal to 20 μm.

In order to conduct the experiment, the HSC-3 cells were synchronized into G_1_ phase by serum starvation and were released into fresh complete medium just before the SERS investigation. This was done to achieve a uniform biomolecular environment around the targeted AuNCs and avoid any cell phase-depended spectral features.^5^ Before UV light exposure, initial SERS spectra of the cell (G_1_ phase), which is selected for this study, were taken to confirm the spectral reproducibility (Fig. S2†). Subsequently, the cells were exposed to the UV light (254 nm) and SERS spectra were collected from a single cell in a time dependent manner. The UV-C light source (254 nm (554.38 μW cm^–2^)) used in this experiment was kept at a distance of ∼5 cm between the lamp and the sample with an angle of ∼45 degree. The live cell chamber was covered with a quartz cover glass (22 × 50 mm) having 0.15–0.25 mm thickness. The sample was continuously irradiated with UV light, and SERS spectra and corresponding DF microscopy images from the same cell were collected at various time intervals (Fig. 1).

Owing to the highly complex nature of the intracellular SERS spectra, only the Raman bands that showed noticeable and consistent modifications were taken into consideration during the spectral analysis. Though the cells did not show any significant spectral modification up to 30 min of UV-C irradiation under these conditions, the SERS spectra showed noticeable changes after 30 min of UV-C exposure. The Raman band, which corresponds to the disulfide vibration (∼502 cm^–1^) gradually decreased in its intensity relative to the intensity of the C–S vibration (∼653 cm^–1^) as UV-C exposure time increased. Apart from this, the vibration at 1000 cm^–1^ (mainly attributed to ring breathing vibration of phenylalanine) and 1027 cm^–1^ (contribution of C–H in-plane bending mode of phenylalanine and the ring breathing vibration of tryptophan (Trp)) also showed noticeable changes. As a result of UV-C induced cellular damage, the broad Raman band found at 990–1030 cm^–1^ split into multiple bands (Fig. 1; after 40 min). This was accompanied with an enhancement in the Raman band found at 1584 cm^–1^ and increased with exposure time. After 80 min, the Raman band found at 1000 cm^–1^ and 1584 cm^–1^ markedly increased, which suggests apoptotic cell death.^23^ Time dependent DF images of the HSC-3 cells taken during continuous UV-C exposure is given in Fig. S3.† These images clearly shows the cell shrinkage and plasma membrane blebbing, which is characteristic of apoptosis. It was also noted that the vibration observed at 1218 cm^–1^, which mainly attributed to the amide III-β conformation of proteins (with contribution from C_6_H_5_–C stretching vibrations of phenylalanine and tryptophan), also increased as a function of exposure time. The features observed at 1130 cm^–1^, which is attributed to C–N vibration of proteins and contributions from the backbone vibrations of lipids, showed a decrease in intensity after 50 min. This spectroscopic observation strongly suggests the possible lipid and protein damage due to the attack of free radicals initiated during UV-C light exposure. It is also important to note that the functionalized AuNC used throughout these studies do not contain amino acid residues of phenylalanine, tryptophan, or tyrosine; therefore, the spectral features experimentally observed can only originate from proteins found within the cellular environment.

In order to rule out the possibility of any laser induced cell damage and subsequent spectral modifications during this study, we did control experiments, where the SERS spectra were collected from the HSC-3 cells without exposing them to the UV light while keeping all other parameters constant. Nearly consistent spectra, without any significant spectral modifications were obtained in this case (Fig. S4†). This result clearly suggests that laser-induced cell damage is unlikely under these experimental conditions and the observed spectral modifications during UV exposure is merely due to the damage caused by UV irradiation.

**Fig. 2:**
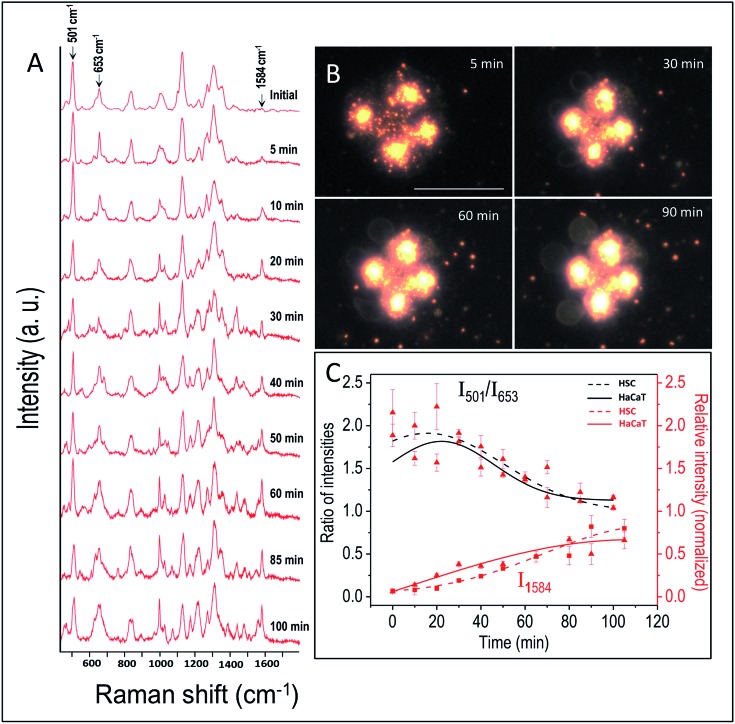
(A) SERS spectra collected from the HaCaT cells while they are exposed to UV-C light (254 nm). (B) DF microscopy images collected at various time intervals, when the HaCaT cells were exposed to UV-C light. (C) Ratio of intensities of I_501_/I_653_ and normalized intensity of vibration corresponds to 1584 cm^–1^ band (I_1584_) collected as a function of time when the HaCaT and HSC-3 cells were exposed to UV light (254 nm). Scale bar shown in (B) and applicable to all DF images is equal to 50 μm.

By following the same method used in the case of HSC-3 cells, UV-C light-induced biomolecular modifications in non-cancerous HaCaT cells were probed using SERS. The Raman spectra collected as a function of time during the UV-C exposure is given in Fig. 2A. The DF images collected at 5, 30, 60, and 90 min of UV-C exposure are also given in Fig. 2B. The spectral trends showed noticeable similarities in both the cases (HSC-3 and HaCaT) as the ratio of intensities of I_501_/I_653_ showed gradual decrease upon UV-C exposure after ∼40 min and the normalized intensity of vibration corresponding to the 1584 cm^–1^ vibration band (I_1584_) increased with time (Fig. 2C). However, Raman spectra and DF images collected from HaCaT cells showed early signatures of apoptosis. A distinct Raman band characteristic of apoptosis appeared after 10 min of UV exposure to HaCaT cells. At the same time, HaCaT cells showed membrane blebbing after 30 min of UV-C exposure, which is a morphological characteristic of apoptosis and was clearly observed in the DF images. These observations point toward the vulnerability of HaCaT cells compared to HSC-3 cells towards the UV-C light induced cellular damage.

**Fig. 3:**
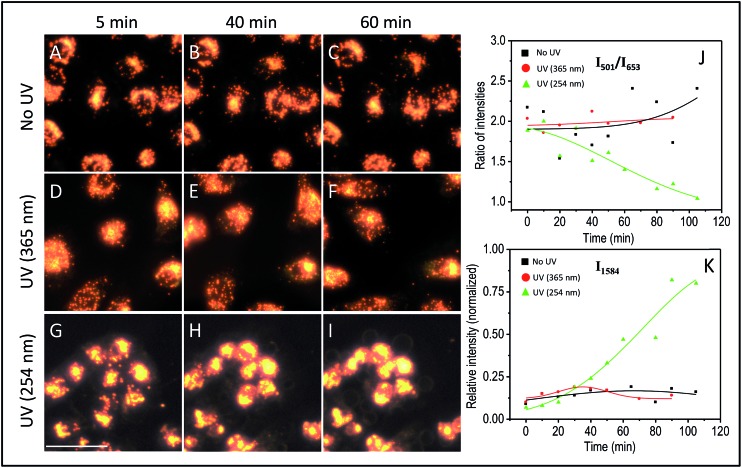
DF microscopy images of HSC-3 cells taken under normal (A–C) conditions, UV light exposure of 365 nm (D–F), and 254 nm (G–I). Ratio of intensities of I_501_/I_653_ (J) and normalized intensity of the vibration corresponding to the band at 1584 cm^–1^ (I_1584_) (K) collected as a function of time when the cells are subjected to different conditions such as absence and presence of UV lights (254 nm and 365 nm) exposure. The scale bar shown in the image G and applicable to all DF images is equal to 50 μm.

The effect of UV-C exposure on the transport of AuNCs within the cytoplasm of cells and cellular motion were also analyzed in real-time using DF microscopy (see DF images shown in Fig. 1 and ESI† video file Video S1). From 0–∼30 min of UV-C exposure, the cells and AuNCs contained inside the cell cytoplasm showed constant, directed motion, which was probed using the strongly enhanced light scattering property of targeted AuNCs inside the HSC-3 cells. After ∼40 min of UV-C exposure, both cellular motion and AuNC motion were completely arrested. This was also observed in the case of HaCaT cells. The cells appeared to be in a ‘*frozen*’ state (Fig. 1 and Video S1†). This was accompanied with significant modifications in the cellular SERS spectra, which clearly indicate the photolytic damage of proteins responsible for intracellular transport. However, in absence of UV light, the cells showed normal behavior mobility during the course of the experiment and the AuNCs located inside cells showed constant, directed motion (Video S2 in ESI†). Plasma membrane blebbing,^24^ characteristic of apoptosis, also started appearing after 40 min (clear in the large area DF image given in Fig. 3) of UV-C exposure. This was not seen in the absence of UV light (Fig. 3). DF microscopic images and the ratio of intensities of S–S and C–S vibration (I_501_/I_653_) as well as normalized intensity of vibration corresponds to 1584 cm^–1^ band (I_1584_) were collected as a function of time in the absence and presence of UV light exposure (254 nm and 365 nm) are given in Fig. 3. The amount of membrane blebbing increased as a function of UV-C irradiation time. A drastic decrease in the I_501_/I_653_ ratio and simultaneous increase in the I_1584_ was distinct after 40 min UV-C irradiation (Fig. 3J and K).

The observed Raman spectral changes can be largely attributed to UV-C irradiation-induced permanent protein damage. It has been shown that UV-C can potentially damage proteins either by modifying protein cysteinyl thiol groups through photolysis or by generate reactive species.^25^ Here, photo-excitation of aromatic amino acid residues such as tryptophan can induce disruption of the disulfide bond in proteins by the transfer of an electron from the excited aromatic residue to the disulfide bond.^26^ The observed reduction in the intensity of the disulfide vibration may be attributed to the photolytic disulfide bond rupturing. This can result in permanent chemical and biological damage as well as enzyme inactivation.^27^ Even after long time exposures to UV-C, the disulfide vibration did not completely disappear as the photolytic cleavage of S–S bond results only by the excitation of aromatic amino acid residues (*e.g.* tryptophan) typically located next spatial neighbors to disulfide bridges.^28^ Apart from that, UV photolysis can induce the photo-ionization and oxidation of aromatic amino acids.^29^ The observed splitting in the molecular vibrations corresponding to the phenylalanine and tryptophan may be caused from their photo-oxidized products. As the length of UV exposure time increased, the intensity of the phenylalanine-based vibrations also increased. This observation could possibly be due to the protein conformational alterations from protein denaturation and subsequent exposure of hydrophobic amino acid moieties to the nanoparticle surface, which is constant with characteristics of apoptosis.^23^ An enhancement in the vibration that is mainly due to the amide III-β conformation (∼1218 cm^–1^) also supports this finding.

As a result of the direct photolytic damage to the proteins inside the cell, it is likely that the free thiol formed by the UV light-induced disulfide rupturing may get adsorbed on the AuNCs.^26^,^30^ It has been shown that continuous exposure of UV light can promote the reduction of large number of disulfide bonds in proteins so as to enhance the free sulfhydryl contents.^30^,^31^ Strong interaction of proteins located in the cytoplasm onto the AuNC surface through these free sulfhydryl groups may inhibit the active protein transport and subsequent movement of Au nanoparticles inside the cell as well as arresting other cellular activities (as observed in the real-time DF images and SERS spectra). A pictorial representation of the aforementioned mechanism is given in Fig. S5A.†

**Fig. 4:**
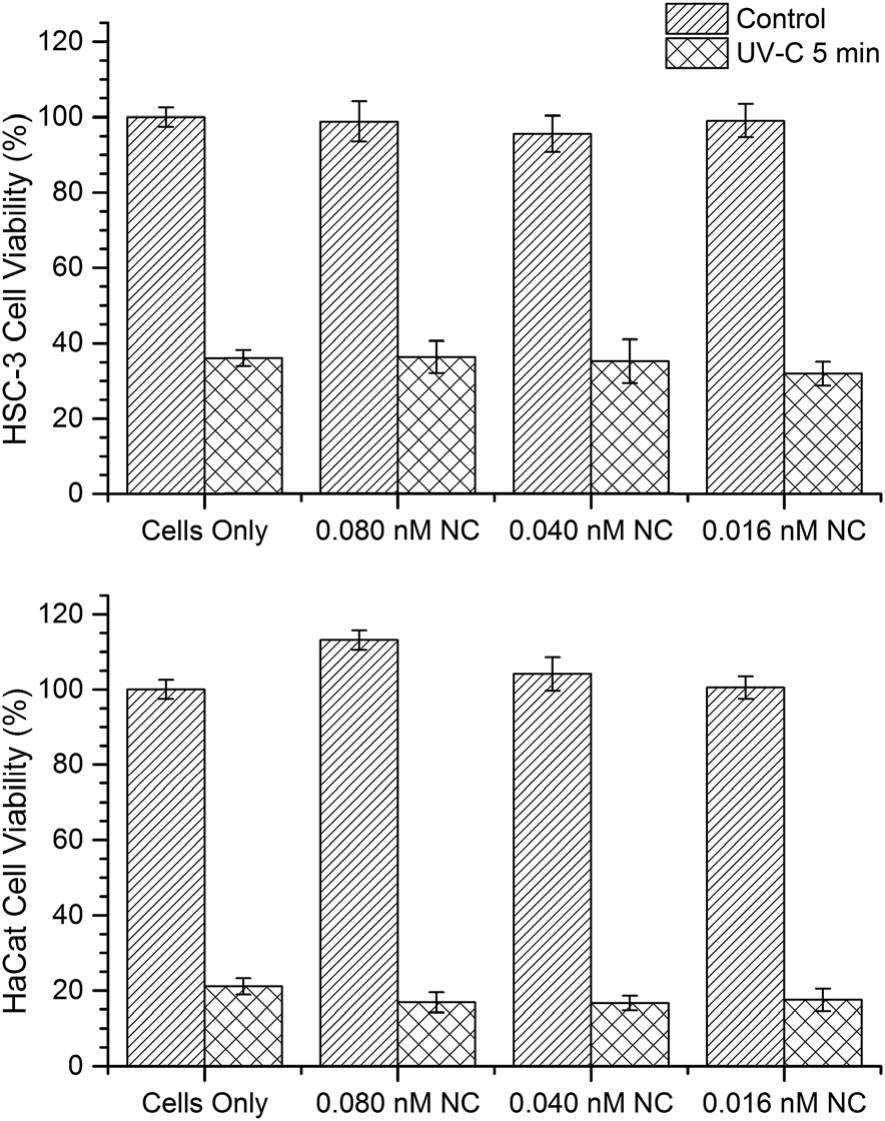
Cell viability of HSC-3 and HaCaT cell lines were performed by evaluating mitochondrial activity through an XTT cell viability assay. UV-C treated (5 min) cells are less viable than non UV-C treated cells regardless of AuNC concentration. Error bars represent standard error.

In order to understand whether the internalized AuNCs might influence (reduce or enhance) the extent of UV irradiation-induced cell damages, we investigated the cell viability through a mitochondrial activity assay (XTT assay) on both HSC-3 and HaCaT cells dosed with 0.08 nM AuNC with and without exposure to UV-C light for 5 min (Fig. 4). In addition, control experiments were performed with both cells lines without the AuNC treatment. A significant reduction in the cell viability was observed in both cell lines when exposed to UV-C in the presence and absence of AuNC (Fig. 4). This experimental result can rule out any significant enhancement or reduction in UV-C radiation-induced cellular toxicity caused from the AuNCs.

**Fig. 5:**
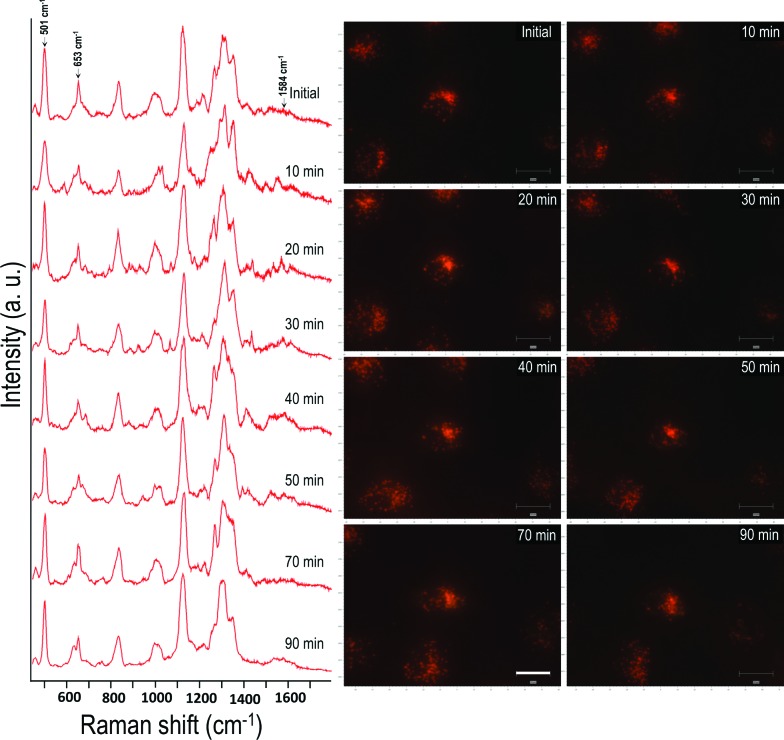
Time dependent SERS spectra collected from a HSC-3 cell exposed to UV-A light (365 nm) in real-time. Corresponding DF microscopy images collected at respective time intervals are also shown. Scale bar is equal to 20 μm.

Next, we investigated the role of UV irradiation in producing free thiols by the photolytic cleavage of dithiols in proteins and their subsequent interaction with AuNCs. Here, we used bovine serum albumin (BSA) as a model protein for our studies. Ellman's assay was used for the estimation of free thiol groups in BSA. 0.5 mM of AuNCs dissolved in 5 mM of 5 mL BSA solution (in PBS buffer) was incubated for 40 min. The solution was then irradiated by UV-C light for 40 min. The resultant solution was centrifuged and the free thiol present in the supernatant was quantified using the Ellman's test. As a control experiment, 0.5 mM of AuNCs dissolved in 5 mM of 5 mL BSA solution (in PBS buffer) was incubated for 40 min. The solution was then centrifuged and the supernatant without AuNCs was irradiated with UV-C light for 40 min. A considerable reduction in the amount of free thiol was observed when the BSA solution was irradiated with UV-C light in presence of AuNCs (Fig. S5B†). This clearly indicates the strong interaction of AuNCs with the free thiols formed by the UV-C photolysis of proteins contained within the cytoplasm and subsequent possibility of locking protein dynamics and transport in a ‘*frozen*’ state. Furthermore, the role of UV-C light in inducing photolytic damage to proteins contained in the cytoplasm was confirmed by investigating the wavelength dependence of UV damage. Though UV-A (365 nm) light is harmful to cells, the possibility of disulfide rupturing is unlikely due to the insufficient energy of UV exposure to photochemical reaction to occur. As expected, UV-A irradiation (365 nm (713.74 μW cm^–2^)) did not show any drastic modification in the SERS spectra of the HSC-3 cells even after 90 min of constant irradiation (Fig. 5). The ratio between the S–S vibration and C–S vibration remained nearly the same throughout the entire irradiation time and the cells did not display any apoptotic signatures, such as membrane blebbing (Fig. 3 and 5). Apart from this, the AuNC motion inside the cells as well as general cellular motion, was not arrested or in a ‘*frozen*’ state. This observation after 90 min of UV-A irradiation rules out any significant disulfide rupturing or photolytic protein damage. The SERS spectra and DF image collected from the HSC-3 cells while exposed to 365 nm UV light are given in Fig. 5 (also see Video S3 in ESI†).

**Fig. 6:**
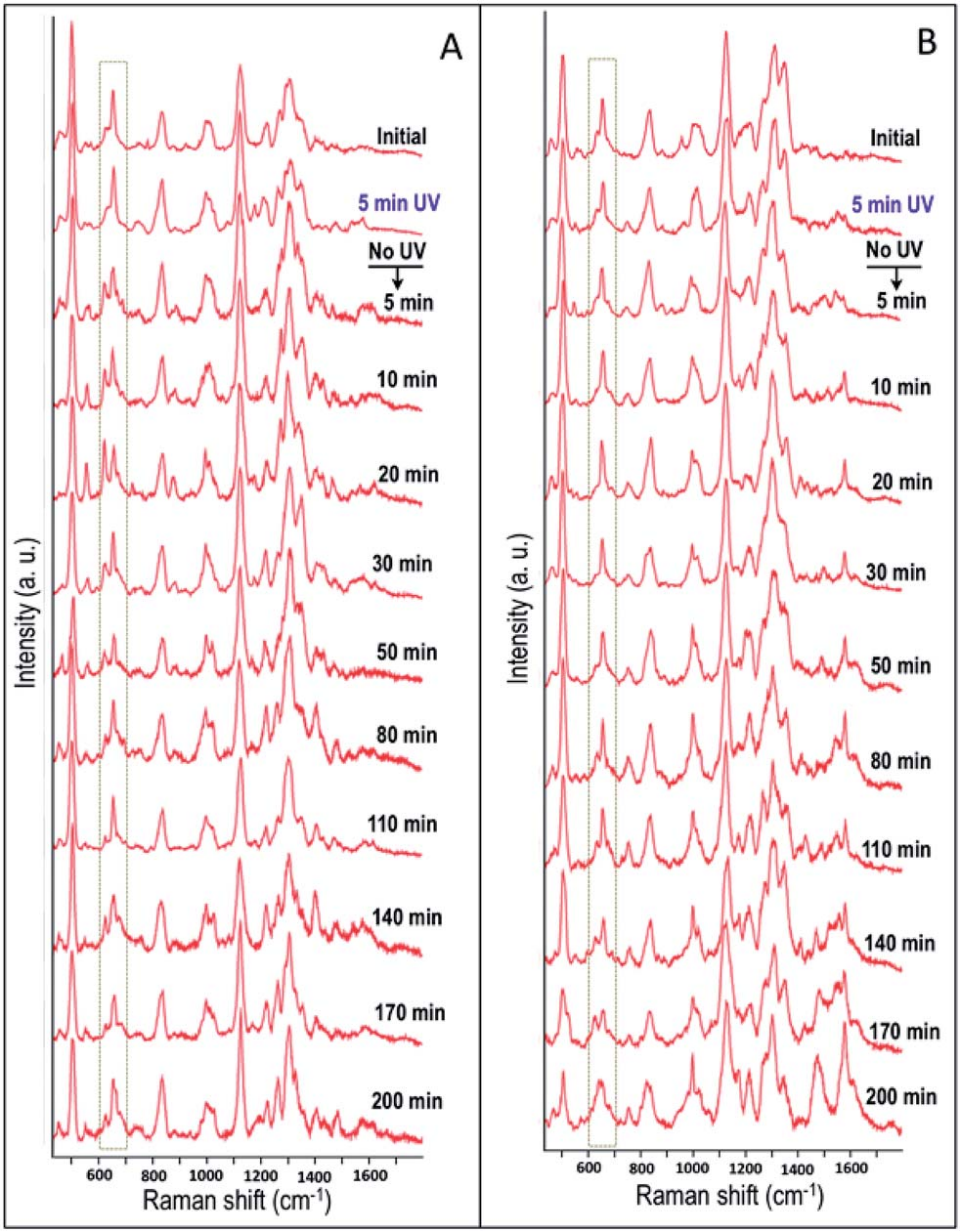
Time dependent SERS spectra collected from (A) HSC-3 cells and (B) HaCaT cells, exposed to UV light (254 nm) for 5 min, followed by only SERS monitoring.

In order to study the cellular response to external stimuli in cancerous and non-cancerous cells, a temporary mutation to the cells were induced by exposing them to the UV-C light for 5 min. Similar to the previous studies, the cells were previously synchronized to the G_1_ phase. Afterwards, the influence of UV-C on the cellular behavior in HSC-3 and HaCaT cells were monitored by collecting the SERS spectra in real-time (Fig. 6). The spectral profile for the two different cell lines showed significant differences under these experimental conditions. Interestingly, the Raman band that corresponds to the C–S stretching vibrations (∼653 cm^–1^) in HSC-3 cells showed a distinct modification after the UV-C exposure. This band appeared as a broad band in the spectrum as the dihedral angle of the C–S–S–C bonds in proteins could exist in different conformations.^32^,^33^ After 10 min of UV irradiation, a shoulder to the main Raman band appeared at ∼621 cm^–1^ and gradually increased in intensity until a maximum was reached after 20 min of exposure (Fig. 6A). Upon reaching peak intensity, this band gradually reduced with respect to time. The Raman band found at 621 cm^–1^ can be mainly attributed to the C–S stretching vibration. Ultraviolet radiation is known for stimulating the production of ROS inside the cells.^34^ As a consequence of the oxidative stress, the cell activates chemical pathways to increase antioxidant levels. Among the antioxidants involved in the maintenance of the intracellular redox balance, reduced glutathione (GSH) plays an important role in antioxidant defense machinery as well as metabolic processes.^35^ GSH, a tripeptide made up of glycine, glutamic acid, and cysteine, is the most abundant thiol containing compound existing in millimolar concentrations (0.1 to 10 mM) in most cell lines, which constitutes the largest component of the endogenous thiol buffer.^36^,^37^ The Raman band at ∼621 cm^–1^ is assigned to the molecular vibration corresponding to the GSH formed inside the cells as a protective response of the cell to oxidative stress generated by the UV-C exposure. It is known that elevated GSH levels are observed in various cancer cells, where high GSH levels promote cancer cell survival.^38^

**Fig. 7:**
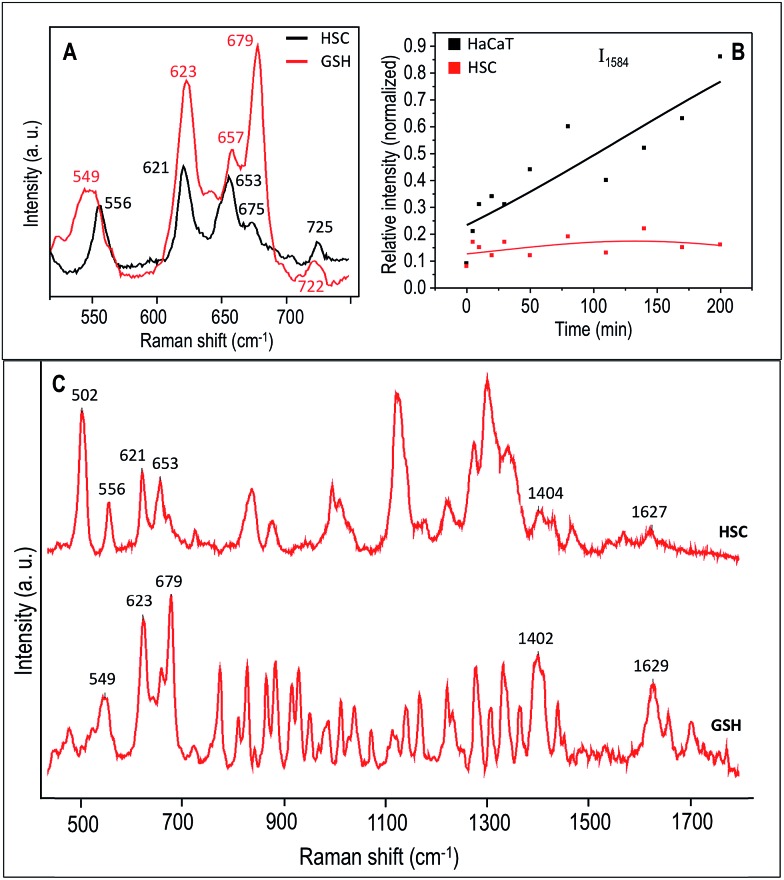
(A) A selected region of the SERS spectrum representative of HSC-3 cells (black trace), which was exposed to UV-C light for 5 min, and collected after 20 min. The corresponding region of the Raman spectrum for GSH is also shown (red trace). (B) Plot showing the normalized intensity of the vibration corresponds to 1584 cm^–1^ collected as a function of time after 5 min UV-C exposure. (C) Large area SERS spectra over the entire spectral range of GSH and HSC-3 cells, which was exposed to UV-C light for 5 min, and collected after 20 min.

To further investigate the extent of GSH contribution to the Raman band observed at 621 cm^–1^, we obtained the Raman spectra of a pure GSH in its reduced form. The C–S vibration region of the Raman spectrum collected from the HSC-3 cells, which is pre-exposed to UV-C light for 5 min, followed by 20 min of no UV exposure and the spectrum of pure GSH is shown in Fig. 7A under 785 nm excitation. A large area spectrum over the entire spectral range is given in the Fig. 7C. The spectral features found in the HSC-3 spectrum after 20 min showed strong resemblance to the corresponding spectrum of GSH. Even though the spectral position showed slight shifts, the Raman vibrations found at 556, 621, 653, 675, and 725 cm^–1^ were also present in the Raman spectrum of GSH, which strongly suggest that the observed vibration found at 621 cm^–1^ could be mainly due to excess GSH molecule formed during the UV-C exposure. These features are in agreement with earlier published results.^39^ In addition to this clear distinction, many other Raman features were also common in both the spectra (Fig. 7C). Details on the assignments of the main Raman vibrations in the SERS spectra are given in Table S1 (Fig. S6†).

In the Raman spectrum of HSC-3 and HaCaT cells in the G_1_ phase, the vibration attributed to GSH found at 621 cm^–1^ appeared as a shoulder band along with the main C–S vibration of the protein moiety. At the same time, such modifications were absent in the case of HaCaT cells, which are exposed to the UV-C light (254 nm) for 5 min under the same conditions (Fig. 6B). After 10 min of UV exposure, the vibrations corresponding to phenylalanine found at 1000 cm^–1^ and 1584 cm^–1^ gradually increased in intensity, which can be attributed to the features corresponding to the activation of apoptotic pathways (Fig. 7B). Even though these bands showed slight modifications after 15 min of UV-C exposure as in the case of HSC-3 cells, no further damage was observed. The spectral modifications were momentary and the cells did not exhibit any morphological signs of apoptosis even after 3 h. However, the HaCaT cells did not recover from the damage caused by the 5 min UV-C light exposure and underwent apoptosis, which was clear from the SERS spectra and DF images. Cell membrane blebbing was obvious in the DF images after 30 min. These results point towards the fact that a high percentage of GSH in the HSC-3 cells promotes its survival rate compared to HaCaT cells towards UV-C-induced oxidative stress. Whereas, low GSH levels in HaCaT cells make them more susceptible towards oxidative stress and subsequent apoptosis. This was further confirmed with the XTT assay, where the percentage of cell viability after UV-C exposure for 5 min was higher in HSC-3 cells (Fig. 4). This is in agreement with earlier reports, which showed that UV light can induce severe GSH depletion in keratinocytes and can enhance the susceptibility to UV-induced damage.^40^,^41^

Momentary disappearance of these features suggest that, although GSH generated inside the cytoplasm is within close proximity to the AuNC surface, the GSH had not yet chemically interacted with the AuNCs. If GSH interacts with the AuNC surface through a permanent thiol–Au covalent interaction, the Raman feature attributed to the GSH would be constant throughout the time course of the experiment, further corroborating its ability to be involved in dynamic cellular pathways. However, HSC-3 cells did not show any morphological signatures of apoptosis and appeared healthy under DF imaging and SERS analysis even after 3 h. This indicates that it is likely the newly formed GSH after 5 min UV-C irradiation interacts with nearly formed ROS. This observation points toward the fact that elevated GSH levels in various tumors make them more resistant to treatments such as chemotherapy.

## Conclusions

Cellular responses of cancer cells towards various UV irradiations were elucidated in real-time using targeted SERS and DF microscopy. Using these techniques, we have probed the UV-C light-induced photolytic cleavage of disulfide bonds present in intracellular proteins contained within the cytoplasm and subsequent deactivation of protein activity and induction of apoptotic cell death. The enhanced cellular defense mechanism by cancer cells was also probed using this technique. We found that, while momentary exposure of UV-C light induces apoptotic cell death in healthy cells, this momentary exposure triggers the glutathione mediated defense machinery in cancer cells in order to recover from the oxidative stress-induced by the UV-C light. We probed the involvement of glutathione during this process in real-time using SERS. These results provide new insights in understanding the mechanism of thiol metabolism associated with cellular defense against externally applied cellular stress.

## Experimental section

### (a) Materials

Hydrogen tetrachloroaurate trihydrate (HAuCl_4_·3H_2_O), sodium borohydride (NaBH_4_), ascorbic acid, cetyltrimethylammonium bromide (CTAB), reduced glutathione (GSH), 5,5′-dithiobis-(2-nitrobenzoic acid) (DTNB), and trisodium citrate were purchased from Sigma-Aldrich USA. Custom-made peptides such as nuclear localization signal, NLS (CGGGPKKKRKVGG), and cell penetrating peptide, RGD (RGDRGDRGDRGDPGC), were procured from GenScript USA, Inc. Thiol-modified methoxypolyethylene glycol (mPEG-SH, MW 5000) was obtained from Laysan Bio, Inc.

### (b) Instrumentation

Transmission electron microscopic (TEM) images of gold nanocubes (AuNCs) were collected using a JEOL 100CX-2 microscope and the average size of the AuNC was determined using ImageJ software. DF images and SERS spectra from the human oral squamous cell carcinoma (HSC-3) and human keratinocyte (HaCaT) cells were collected using a Renishaw inVia Raman Microscope coupled with Leica DM2500M microscope. A 785 nm diode laser was used for the SERS measurements.

### (c) Synthesis of gold nanocubes (∼37 nm edge length)

AuNCs were synthesized by following the modified seed-mediated method reported by Murphy *et al.*^42^ In a typical synthesis, seed nanoparticles were prepared by the reduction of 2.75 mL HAuCl_4_·3H_2_O (0.909 mM) with CTAB solution (0.283 g in 5 mL deionized water (DI)) by an ice-cold 0.01 M NaBH_4_ solution (600 μL) under stirring for 2 minutes. After 1 h, 0.35 mL of tenfold diluted seed solution was allowed to grow overnight in a growth solution, which is prepared by mixing CTAB solution (2.916 g in 400 mL DI water) with HAuCl_4_·3H_2_O (0.0394 g in 143 mL DI water) followed by the addition of 6 mL ascorbic acid (1 M). The resultant AuNCs solution was purified by centrifugation at 9500 × *g* for 10 min followed by redispersion in DI water.

### (d) Preparation of PEG/RGD/NLS-functionalized gold nanocubes

The purified AuNCs were first conjugated with mPEG-SH by incubating them (10 mL of 1.56 nM) with 123.22 μL of mPEG-SH (1 mM) for 24 h and were purified using centrifugation at 9500 × *g* for 10 min. Afterwards, these PEGylated AuNCs (9 mL of 1.62 nM) were treated with 4.14 μL of cell penetrating peptide, RGD (5 mM), and 10.65 μL of nuclear localization signal, NLS (5 mM), to yield PEG/RGD/NLS-functionalized AuNCs. The functionalized AuNCs were purified using centrifugation at 9500 × *g* for 10 min to remove unbound ligands and were redispersed in DI water for subsequent use.

### (e) Cell culture

HSC-3 and HaCaT cells were cultured in Dulbecco's modified Eagles' medium (DMEM, Mediatech), which contains phenol red, supplemented with 10% v/v fetal bovine serum (FBS, Mediatech) and 1% antibiotic-antimycotic solution (Mediatech) in a 37 °C, 5% CO_2_ humidified incubator. For the SERS studies, the cells were grown on glass coverslips in complete growth medium in the incubator at 37 °C for 24 h. Subsequently, the cells were treated with 0.08 nM PEG/RGD/NLS-functionalized AuNCs, diluted in supplemented DMEM cell culture medium, for 24 h. The cells were then synchronized in the G_1_ phase by serum starvation for 24 h as reported earlier.^5^ Afterwards, the cells were released into complete medium and grown for ∼1–3 h before SERS experiments.

### (f) *In vitro* SERS measurements

SERS spectra were collected from the cells under the UV exposure in a time dependent manner. Data collected from 3 independent experiments were analyzed. The spectra were measured from individual cells with a 1200 lines per mm grating using a Renishaw InVia Raman spectrometer. During the spectral measurements, the laser (785 nm) was directed into a microscope and it was focused onto the sample by a 50×/0.75 N. A. objective. The laser power was kept constant for all the experiments (around 6.2 mW). The back-scattered signals from the samples (with an integration time of 10 s) were collected by a CCD detector in the range of 400 to 1800 cm^–1^. A cubic spline interpolation is used for the baseline fit by manually selecting the points representative of the background. DF microscopy images were collected using Lumenera's infinity2 CCD digital camera.

### (g) XTT cell viability assay

Cell viability assays were purchased from Biotium, Inc. and used as per manufactures instructions on cells grown under the same conditions as stated above.

## Supplementary Material

Supplementary information

Supplementary movie

Supplementary movie

Supplementary movie
