# Supplementary material for: Elucidation of ultraviolet radiation-induced cell responses and intracellular biomolecular dynamics in mammalian cells using surface-enhanced Raman spectroscopy
Source: Chem Sci. 2015 Nov 5;7(2):1133–41. doi: 10.1039/c5sc03817k (PMC5975792; doi:10.1039/c5sc03817k)
Supplement: Supplementary file 1 [file SC-007-C5SC03817K-s001.pdf]

Electronic Supplementary Material (ESI) for Chemical Science.
This journal is © The Royal Society of Chemistry 2015

Electronic Supplementary Information

Elucidation of ultraviolet radiation-induced cell responses and intracellular biomolecular dynamics in mammalian cells using surface-enhanced Raman spectroscopy

*Sajanlal R. Panikkanvalappil[a], Steven M. Hira[a], and Mostafa A. El-Sayed[a,b]**

[a]Laser Dynamics Laboratory, School of Chemistry and Biochemistry, Georgia Institute of Technology, Atlanta, Georgia 30332-0400, United States.

[b]King Abdulaziz University, Department of Chemistry, Jeddah 22254, Saudi Arabia.

Electronic Supplementary Information 1

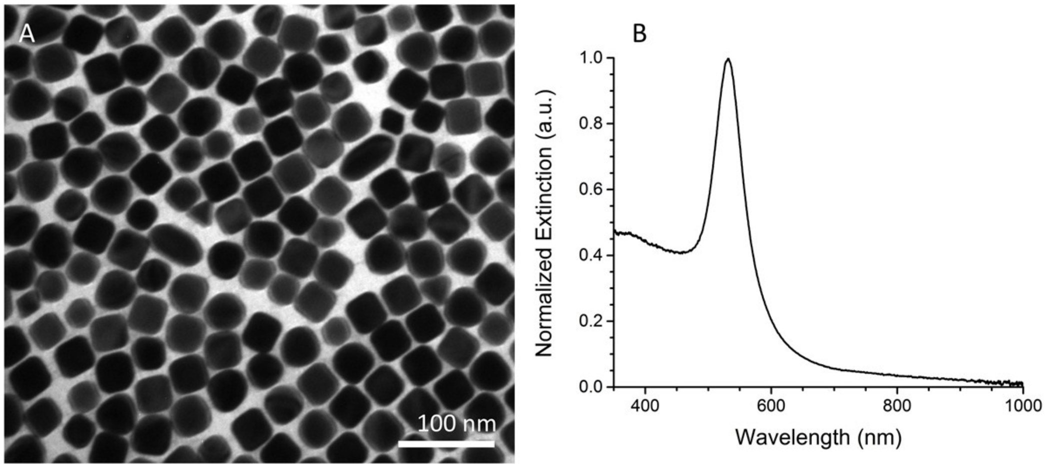

Figure S1. TEM image (A) and corresponding normalized extinction spectrum of AuNCs (B).

Electronic Supplementary Information 2

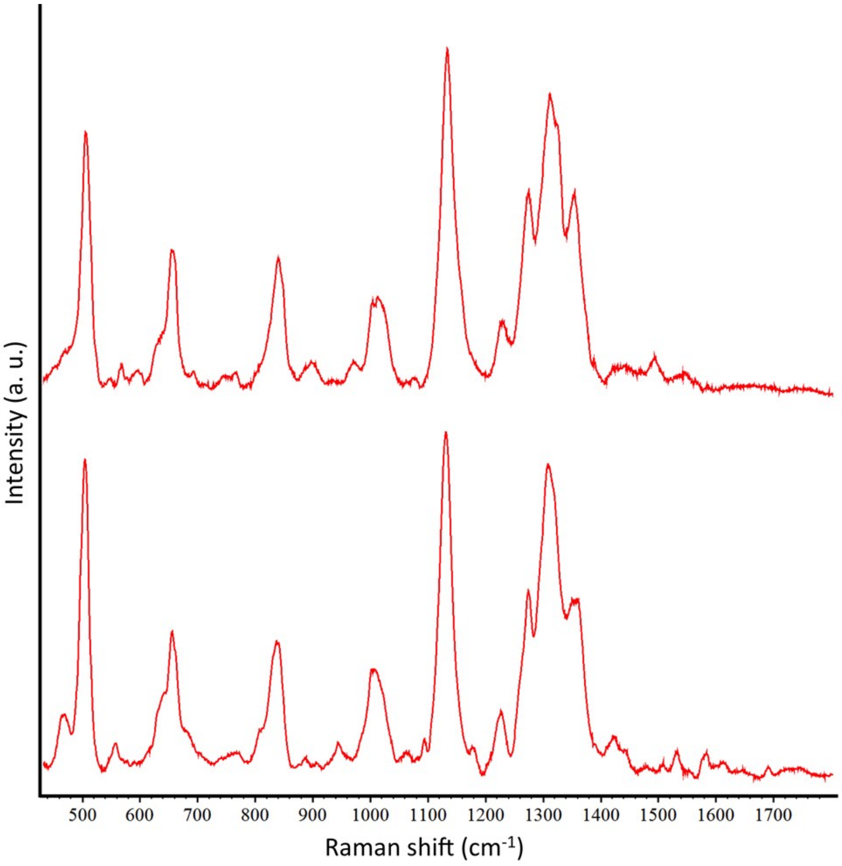

Figure S2. Initial SERS spectra collected from the same HSC-3 cell (G_1 phase) before UV light exposure (5 min interval).

Electronic Supplementary Information 3

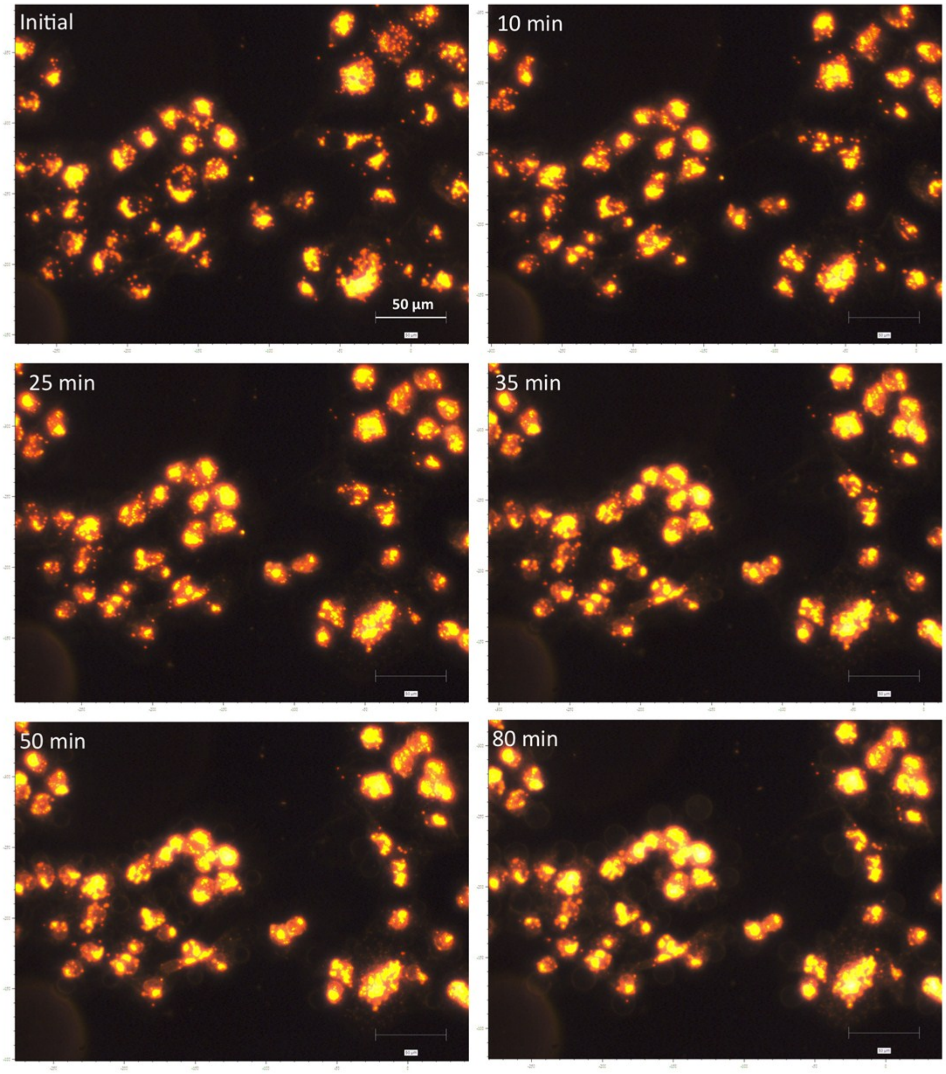

Figure S3. Time dependent DF images collected from a HSC-3 cell while being exposed to UV-C light (254 nm). Scale bar shown panel 1 (initial) applicable to all DF images.

Electronic Supplementary Information 4

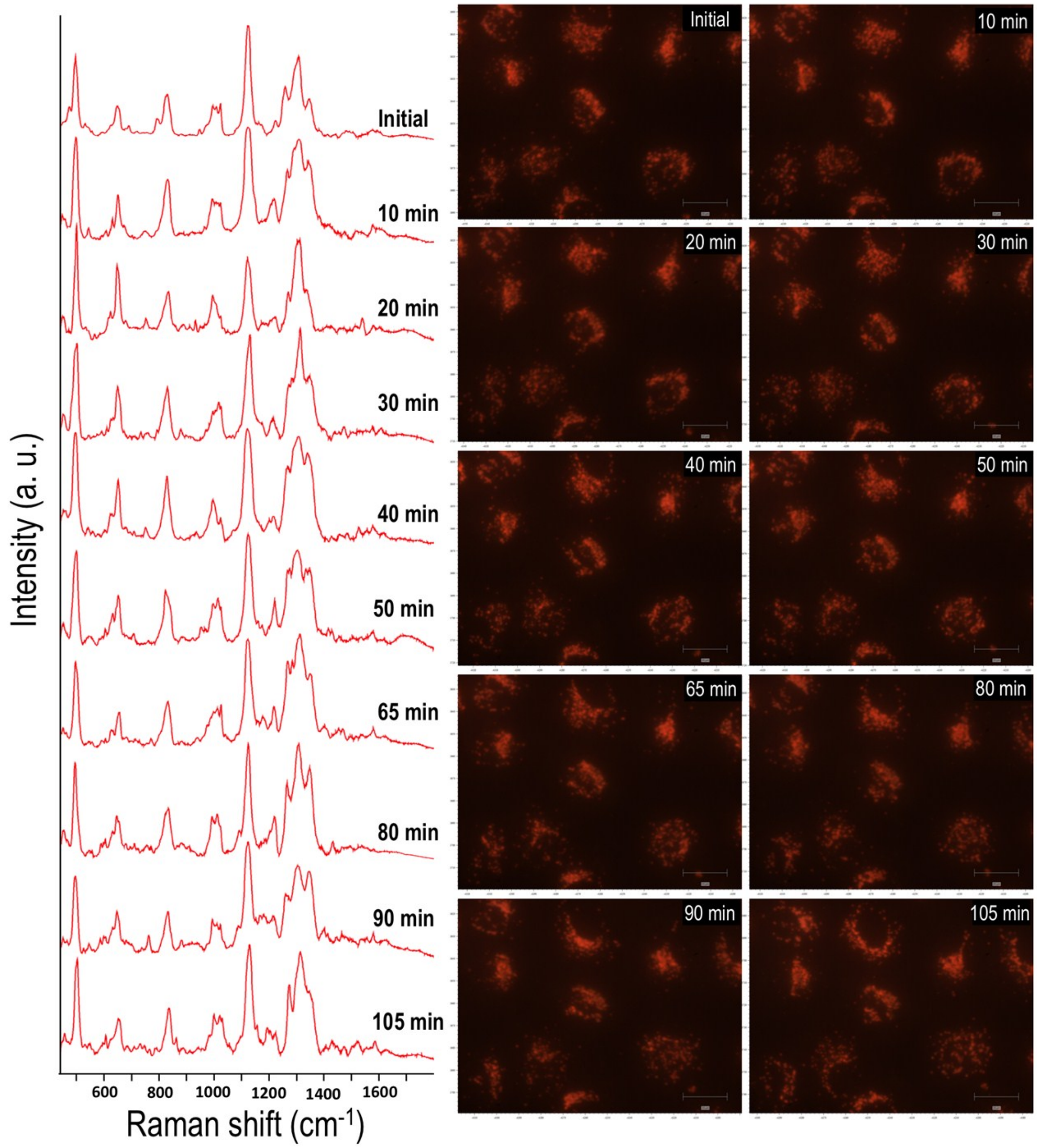

Figure S4. Time dependent SERS spectra collected from HSC-3 cells in absence of UV light. Corresponding DF microscopy images collected at respective time intervals are also given. The scale bars shown in the images are 20 µm.

Electronic Supplementary Information 5

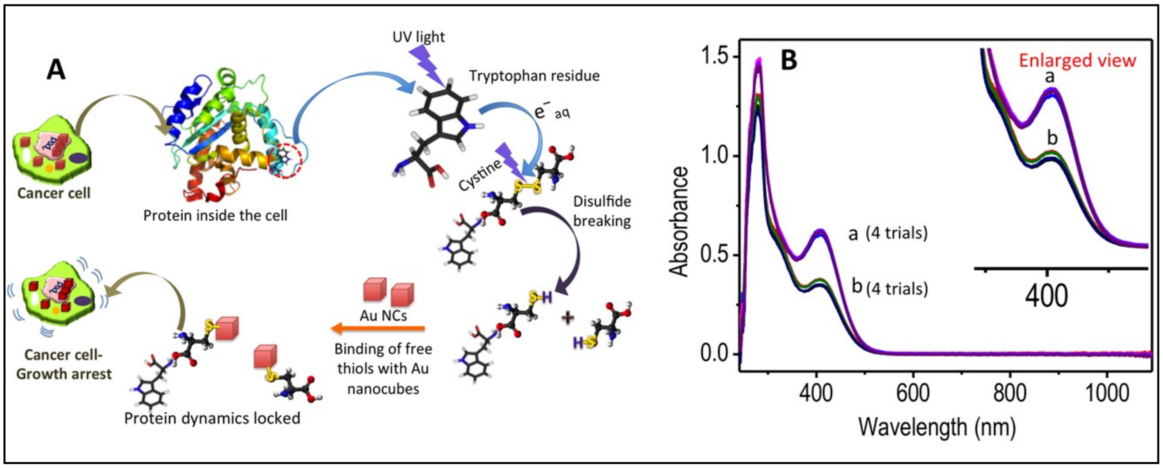

Figure S5. (A) Pictorial representation of the plausible mechanism of UV-light induced disulfide breaking inside cells and subsequent interactions of proteins with AuNCs. (B) UV-vis absorption spectra for (a) BSA + AuNCs solution centrifuged after 40 min. Subsequently, the supernatant was irradiated with UV light for 40 min and Ellman's reagent added to the supernatant. (b) BSA+AuNCs solution exposed to UV light for 40 min and Ellman's reagent added to the supernatant.

Electronic Supplementary Information 6

Table S1. Assignment of bands in the SERS spectra

Wavenumber (cm^{-1})	Component	Tentative assignments of SERS bands
495-510	Protein	-S-S-
620-660	Protein	-C-S-
800-850	Proteins and lipid	Tyrosine in proteins and C_4N^+, O—C—C—N symmetric stretches in lipids
1000-1010	Protein	RB vibration of Phe
1012-1035	Protein	C-H in-plane bending mode of Phe and RB vibration of Trp
1100-1140	Lipid and protein	C-N vibration of proteins and Gauche and all-trans conformations of lipids
1200-1235	Protein	C_6H_5–C stretching vibrations of Phe and Try, amide III (β-pleated sheet)
1240-1255	Protein	Amide III (random coil)
1265-1300	Protein	Amide III (α-helix)
1300-1325	Lipid	$-CH_2$ twist
1440-1460	Protein and lipid	CH_2 bending mode of proteins and lipids along with methylene deformation
1584	Protein	Phe

RB=Ring breathing; Try=Tryptophan; Phe=Phenylalanine
